# Switch from sexual to parthenogenetic reproduction in a zebra shark

**DOI:** 10.1038/srep40537

**Published:** 2017-01-16

**Authors:** Christine L. Dudgeon, Laura Coulton, Ren Bone, Jennifer R. Ovenden, Severine Thomas

**Affiliations:** 1 The University of Queensland, Molecular Fisheries Laboratory, School of Biomedical Sciences, St. Lucia Queensland, 4072, Australia; 2 Reef HQ Aquarium, Townsville, Australia; 3 College of Marine and Environmental Sciences, James Cook University, Townsville, 4811, Queensland, Australia

## Abstract

Parthenogenesis is a natural form of asexual reproduction in which embryos develop in the absence of fertilisation. Most commonly found in plants and invertebrate organisms, an increasing number of vertebrate species have recently been reported employing this reproductive strategy. Here we use DNA genotyping to report the first demonstration of an intra-individual switch from sexual to parthenogenetic reproduction in a shark species, the zebra shark *Stegostoma fasciatum*. A co-housed, sexually produced daughter zebra shark also commenced parthenogenetic reproduction at the onset of maturity without any prior mating. The demonstration of parthenogenesis in these two conspecific individuals with different sexual histories provides further support that elasmobranch fishes may flexibly adapt their reproductive strategy to environmental circumstances.

Parthenogenesis is a natural form of asexual reproduction in which embryos develop in the absence of fertilisation. Occurrences of parthenogenetic reproduction in vertebrate organisms have been increasingly documented (recorded from >0.1% of extant vertebrate species)^1^. Obligate parthenogenesis, where all individuals within a species reproduce asexually, is restricted to the Squamate reptiles^2^,^3^. Facultative parthenogenesis, the occurrence of asexual reproduction in otherwise sexually producing species, is found more widely across major vertebrate groups including reptiles, birds, bony fish and six species of sharks and rays^1^,^3^,^4^,^5^,^6^,^7^,^8^,^9^,^10^,^11^,^12^. Mammals are an exception as facultative parthenogenesis does not naturally occur in this group due to intracellular processes such as genomic imprinting during gametogenesis^13^.

Most documented cases of facultative parthenogenesis in vertebrates have been recorded from females in captive environments that have had no exposure to male conspecifics during their entire reproductive lifetime^3^,^6^. This raises questions regarding the adaptive strategy of facultative parthenogenesis in these isolated incidences or whether parthenogenesis in most vertebrates is accidental^14^. Novel lines of evidence can help elucidate the prevalence and function of parthenogenesis in vertebrates. In particular, parthenogenesis has been demonstrated in wild vertebrate populations: pit viper snakes^15^ and sawfish^8^. Parthenogenetic offspring in these populations were identified among sexually produced offspring based on their unusually high levels of genetic homozygosity. This genetic signature in vertebrates is mostly attributed to the mechanism of terminal fusion automixis, the restoration of diploidy by fusion of the egg with a polar body^12^, although gametic duplication also leads to elevated homozygosity and in most cases cannot be disregarded as the potential mechanism^3^. The presence of sexually produced litters captured from the same regions and time periods as parthenogenetic offspring suggest that complete isolation from males during a female’s reproductive lifetime may not be a requirement or even a driver.

A recent study on a captive eagle ray *Aetobatus narinari* suggests that relatively short periods of separation from a potential mate may trigger a shift in reproductive strategy^9^. A single female eagle ray switched from sexual reproduction to producing a pup asexually less than one year after being separated from the male^9^. Only one other published study demonstrates this switch within an individual vertebrate. A captive *Boa constrictor imperator* produced a litter through a sexual encounter with a co-housed male *B. c. constrictor*. After a four year period of isolation she was housed with other male conspecifics during which she produced two litters. Genetic analyses demonstrated that these were comprised of parthenogenetic offspring despite what appeared to be potential mating opportunities^16^. In three other cases, captive female pythons have produced parthenogenetic offspring after having been observed copulating with male conspecifics. However, the fertility of these male snakes was not determined^3^,^17^.

Here we report on the first occurrence of an intra-individual switch from sexual to parthenogenetic reproduction in a shark species, the zebra shark *Stegostoma fasciatum*. This study is also novel in demonstrating the onset of parthenogenetic reproduction in two individual, co-housed, females with different sexual histories: parthenogenesis following sexual reproduction and without prior sexual reproduction. Zebra sharks are oviparous^7^, reach maturity around 7 years of age, and live to over 35 years in captivity (pers. obs.). In 1999 a wild-captured female zebra shark (*F1*) was introduced to an already captive mature male zebra shark (*M1*) within the Reef HQ Aquarium, Townsville (Australia). The maturity of *F1* was not confirmed, however mating was attempted at this time. The pair were separated and reunited in 2006, and mating commenced at that stage. *F1* started laying eggs in 2008 and successfully produced several litters of viable offspring each year until 2013 (Fig. 1). Following mating in 2012, *M1* was separated permanently from *F1*. Offspring were produced in the breeding season spanning the austral summer (2012/2013) following this final mating event. During the next breeding season (2013/14) *F1* did not produce any eggs. At this time her immature daughter shark (*F2* born in 2009) was introduced into the same tank as her. *F1* started laying eggs again the following season (2014/15). Live embryos were observed in 6 of the 47 eggs and monitored until they were all deceased between 35 and 94 days of incubation. The daughter shark *F2* reached maturity at this time and also started laying eggs, which were visibly distinguishable from her mother’s eggs due to having a slightly smaller size and thinner shell. None of *F2*’s eggs showed embryo development. During the 2015/2016 breeding season, both *F1* and *F2* laid eggs with some embryos visible for both sharks. Three juvenile sharks hatched out between February and April 2016 from the eggs of *F1*, and one juvenile shark hatched out in June 2016 from the eggs of *F2* (Fig. 1).

The presence of the embryos in the eggs of *F1* following the separation from the male could be explained by two hypotheses: (i) storage of *M1*’s sperm by *F1*, or (ii) parthenogenesis. Both hypotheses are plausible. Parthenogenesis has previously been described for this species from one captive zebra shark in the Dubai aquarium with no history of sexual reproduction^7^. *F2* was not housed with reproductively mature males at any point so only the parthenogenesis hypothesis seems plausible in her case. Although the duration of sperm storage has not been investigated in zebra sharks specifically, sperm storage for up to 45 months has been reported from a related carpet shark species^18^ and the longest confirmed sperm storage of any vertebrate is recorded at 67 months in the eastern diamond-backed rattlesnake (*Crotalus adamanteus*)^19^, clearly spanning beyond the period of isolation from a male that *F1* experienced. If sperm storage accounted for the offspring of *F1* in the absence of a mate, the genotypes of the offspring will reflect two-parent origin and reject the hypothesis of parthenogenesis. We employed DNA genotyping to test between these two competing hypotheses and demonstrated that *F1* switched between sexual and parthenogenetic reproductive modes quickly, skipping only one breeding season, while the daughter shark (*F2*) commenced her reproductive phase via parthenogenesis one year after maturity without any exposure to a mate. This study highlights the flexibility in reproductive strategies for elasmobranchs and we discuss the consequent ecological and evolutionary implications.

## Results and Discussion

In total 14 zebra shark specific loci were scored. Nine of these loci demonstrated unique alleles that were not shared between the mother *F1* and putative father *M1* shark, and were therefore informative for parental assessment of the offspring (Table 1). For these nine loci, the offspring from the 2009 and the 2013 (n = 1–3) seasons were presumed to be of sexual origin from *F1* × *M1* and expected to demonstrate bi-parental inheritance. These individuals were heterozygous at all nine loci displaying one maternal and one paternal allele, in accordance with the sexual reproduction hypothesis. The presumed parthenogenetic offspring from *F1* (2015:n = 1–4, 2016:n = 1–3) were homozygous for one of the maternal alleles at each locus. The single offspring of *F2* (2016:5) was homozygous at all alleles that were present in *F2*’s genotype. As *F2* is the sexually produced daughter of *F1*, the alleles from eight of the nine loci also matched *F1*’s genotype. However, one locus (Sfa221) distinguished the mother of this offspring as *F2*. The offspring (2016:5) was homozygous for allele 242, which was recorded from *F2* (242, 248) and *M1* (238, 242) but not *F1* (246, 248) (Table 1).

The other five loci all demonstrated one shared allele between *F1* and *M1*. Although it is not possible to determine the parental origin of the shared allele when present in the offspring genotype, all of the parthenogenetic offspring were homozygous at each of these loci, fitting the genetic signature of parthenogenesis in elasmobranchs. The sexually produced offspring were either homozygous for the parental shared alleles or heterozygous, fitting the genetic signature of bi-parental inheritance (Supplementary Table).

These results unambiguously support the hypothesis that the embryos produced two years after the removal of the male shark were of parthenogenetic origin and not due to sperm storage. The offspring of *F2* also supported a parthenogenetic origin, demonstrating that *F2* commenced reproducing asexually in her second year of maturity. The elevated homozygosity displayed in parthenogenetic genotypes (from *F1* and *F2*) could be the genetic signature of terminal fusion automixis, which is the dominant mechanism for facultative parthenogenesis proposed for vertebrate animals^5^,^12^,^15^. In this mechanism heterozygosity is restricted to the tips of the chromosomes^12^, therefore genetic signatures of randomly screened microsatellite loci tend to demonstrate elevated homozygosity. Alternative mechanisms, including gametic duplication^19^ and spontaneous development of a haploid individual from an unfertilized egg^20^ result in complete homozygosity^21^ and cannot be ruled out^3^. However heterozygosity was observed at one locus for a parthenogenetic zebra shark in the Dubai aquarium supporting the mechanism of terminal fusion automixis in this species^7^. The analysis of *F1*’s earlier offspring born in 2009 and 2013 clearly demonstrates sexual reproduction where the offspring possess at least one allele from *M1* at each locus. This confirms that *F1* switched from sexual to parthenogenetic reproduction within a period of two years.

Van der Kooi and Schwanten^14^ argued that examples of facultative parthenogenesis in vertebrates are likely to be reproductive errors and hence are indicative of accidental parthenogenesis. Under that model, asexual reproduction is rare and sporadic across species and not an adaptive strategy. Our findings suggest otherwise. Firstly we have demonstrated a relatively rapid transition from sexual reproduction to parthenogenetic reproduction in an individual animal that appears to be in response to an environmental change. Parthenogenesis was not documented from a single, isolated individual, but rather two individuals within the aquarium system with different sexual histories. Furthermore, parthenogenesis has been documented in this species from individuals captured from geographically distant locations: the western Pacific Ocean (this study) and the Red Sea^7^. Other elasmobranch and snake species have also demonstrated parthenogenetic reproduction in multiple individuals as well as across successive years in captivity^3^,^6^,^9^,^16^,^17^,^22^. Furthermore, the viability of a vertebrate parthenogenetic offspring has recently been demonstrated in a bamboo shark with a second generation offspring also being produced through parthenogenesis^23^.

A challenge for understanding the adaptive nature of facultative parthenogenesis in elasmobranchs and other vertebrates is identifying the conditions under which it occurs. Heritability in facultative parthenogenesis has been demonstrated for poultry and *Drosophila* spp. (see review ref. 24). However sexual reproduction appears to be the dominant form of reproduction for species demonstrating facultative parthenogenesis^8^,^15^ and therefore, it appears that internal or external cues may lead to the onset of parthenogenesis in these species. Studies in poultry found that viral infections increased the prevalence of parthenogenesis in different species, but that there were no significant effects from feed types, light levels, sex hormones or proximity to conspecifics. Increasing temperature was found to initiate the onset of parthenogenesis in silkworms and increase its prevalence in *Drosophila parthenogenata* (see review ref. 24). In this study, *F1* was kept in the same aquarium throughout minimizing any changes to her external environment. The main trigger for the switch from sexual to parthenogenetic reproduction in *F1* therefore, appears to be the removal of the mate. Similarly, the rapid transition between reproductive strategies by the eagle ray also followed the removal of the mate, supporting the hypothesis that parthenogenesis is a reproductive advantage under conditions of isolation from potential mates^12^. However this cue does not appear to be ubiquitous among vertebrates with contrasting patterns observed in snakes. A female boa constrictor demonstrated a switch from sexual to asexual reproduction, reproducing parthenogenetically in the presence of male conspecifics and not during the two intermittent years when she was housed in isolation^16^,^25^. Although most examples of parthenogenesis for snakes have occurred when females were isolated from mates, parthenogenesis was also documented from two captive regal pythons and one blood python following copulation with male conspecifics^3^,^17^. However the fertility of these male snakes has not been confirmed. To date, examples of parthenogenesis in elasmobranchs in captivity have only been reported from females isolated from males. To better understand the effect of the absence or presence of males on the onset of parthenogenesis, further studies on the genetic signatures of offspring produced from cohoused male and female individuals are also required.

It is not possible to rule out potential cues between the mother and daughter shark triggering the onset of parthenogenesis. However the female zebra shark in the Dubai aquarium was not housed with another zebra shark at any point prior to maturation and commencing parthenogenetic reproduction^7^, therefore lending support more to the absence of a mate rather than the presence of another female as the driver.

Critical densities have been proposed as a driver for the onset of parthenogenetic reproduction within a species^26^. Under this scenario populations can grow to critical levels through parthenogenesis to increase downstream opportunities for mating success. However given that most examples of parthenogenesis in vertebrates from captive environments involve females kept in isolation or with few conspecifics, it is not possible to determine what a threshold would be, if at all it exists. The few examples of parthenogenesis from wild vertebrates demonstrated overall sex ratios near unity^8^,^15^, yet this does not take into account potential spatial segregation during critical mating periods. Empirical studies in captive conditions could be undertaken to ascertain critical levels at higher densities.

The evolutionary function of facultative parthenogenesis may become clearer when mechanisms are understood across a range of taxa, but at the moment it remains debatable. Most obligate parthenogenetic vertebrates arise from hybridization between closely related species, resulting in elevated individual heterozygosity relative to the parental genotypes^11^,^27^,^28^. This is considered adaptive for colonizing new areas where high genetic diversity may provide the necessary genetic tools to adapt to new conditions^29^. Although most obligate parthenogenetic lineages are short lived and therefore considered of greater ecological than evolutionary importance^11^, they may have long-term evolutionary adaptive advantages where back-crossing with sexual species enables genera to expand phylogenetically and geographically^27^. In contrast, facultative parthenogenesis results in greatly reduced genetic diversity and presumably less adaptive advantage in dealing with novel environmental conditions. The accumulation of deleterious mutations (Muller’s ratchet^30^) results in lineages being short lived unless there is the capacity for sexual reproduction. Sexual reproductive competency of parthenogenetic offspring has not yet been demonstrated in vertebrates though it has been recorded from other organisms (e.g. *Drosophila*^31^).

An interesting point of difference in facultative parthenogenesis between elasmobranchs and other vertebrate species is the consequence of the genetic mechanism for sexual determination. Cytogenetic analysis of a subset of elasmobranch species demonstrated XY male heterogamety and XX female homogamety similar to mammals^32^. This contrasts with birds and many reptiles, which demonstrate ZW female heterogamety with ZZ male homogamety. The exception is the basal snake lineages which may produce viable WW female offspring^25^; however see Booth & Schuett^3^ where it is suggested that basal snakes including the Pythons and Boas may actually possess XX/XY sex chromosomes as opposed to the commonly accepted ZZ/ZW system. Facultative parthenogenesis may be particularly advantageous for species having ZZ male homogamety, as it leads to the production of males, which are potential future mates. In elasmobranchs however, all observed viable offspring produced by facultative parthenogenesis are female^6^,^7^,^9^.

Facultative parthenogenesis leading to female offspring may then have the adaptive advantage of a ‘holding on’ mechanism, through maintaining female lineages until potential male mates become available again following immigration. In particular, elasmobranchs are considered to have ancient lineages with many species extending millions of years back in the fossil records^33^. Population genetic analysis of several elasmobranch species has revealed signatures of population bottlenecks associated with glaciation periods^34^,^35^. Facultative parthenogenesis may have assisted populations to survive through these periods of isolation. To address these ideas it’s important to identify more examples of facultative parthenogenesis from the wild. Although the exact mechanisms triggering facultative parthenogenesis currently remain a mystery, the reproductive flexibility it potentially provides for vertebrates may be underestimated for species’ survival and evolution. Examination of contemporary isolated populations as well as empirical studies with captive individuals will help investigate the mechanisms, functions and prevalence of facultative parthenogenesis in vertebrate species.

## Methods

Tissue samples for DNA analysis were collected during husbandry procedures from the mother shark (*F1*); the putative father shark (*M1*); the mature daughter shark *F2* (hatched 2009 from reproduction between the two former individuals); four of the deceased embryos from *F1* in the austral summer 2014/15 season (2015:1–4); 3 hatchlings and 1 deceased embryo from *F1* in the 2015/16 season (2016:1–4); and 1 hatchling from *F2* (2016:5). To assess the timing of the switch between sexual and parthenogenetic reproduction in *F1*, we also sampled three offspring that had hatched but died during juvenile stages from the last breeding season where the female and male were cohoused (2013:1–3). All methods were carried out in accordance with relevant guidelines and regulations following husbandry procedures within the Reef HQ Aquarium, Townsville and with approval by the University of Queensland Animal Ethics Committee (#ZOO/ENT/490/05).

DNA was extracted and genotyped at 14 microsatellite loci developed specifically for zebra sharks (as per refs 36 and 37). Genotypes were scored using Geneious version 9.1.3^38^.

## Additional Information

**How to cite this article**: Dudgeon, C. L. *et al*. Switch from sexual to parthenogenetic reproduction in a zebra shark. *Sci. Rep.* **7**, 40537; doi: 10.1038/srep40537 (2017).

**Publisher's note:** Springer Nature remains neutral with regard to jurisdictional claims in published maps and institutional affiliations.

## Supplementary Material

Supplementary Table

## Figures and Tables

**Figure 1:**
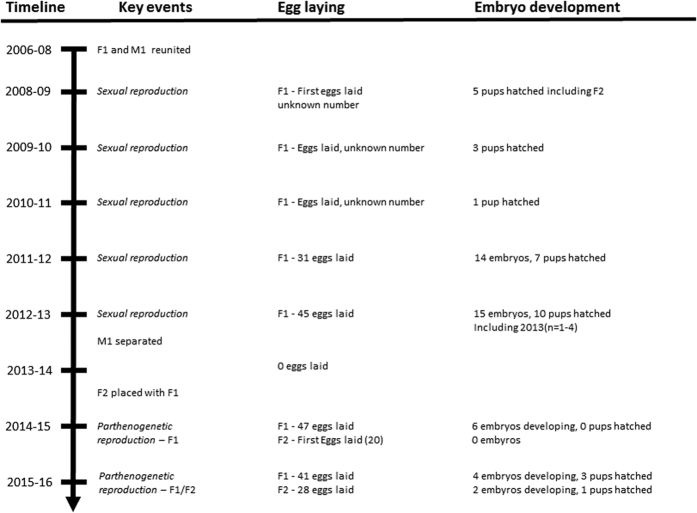
Timeline showing the key events of mating and separation, egg production and embryo development of sexual and parthenogenetic zebra sharks. *F1* refers to the primary mature female and *M1* to the mature male. *F2* is the sexually produced offspring of *F1* and *M2*.

**Table 1:** Genotype data at nine microsatellite loci for 15 zebra sharks *Stegostoma fasciatum* from Reef HQ Aquarium Australia.

Ind.	Description	Parent/s	SF2		SF38		SF72		Sfa221		Sfa236		Sfa248		Sfa335		Sfa387		Sfa418	
F1	Mother		192	194	229	241	238	272	246	248	244	256	229	335	380	400	240	246	231	231
M1	Father		190	190	245	245	222	250	238	242	228	240	307	339	368	372	232	232	225	225
F2 (2009)	Sexual offspring	F1 & M1	190	194	229	245	222	238	242	248	240	256	299	307	368	380	232	240	225	231
2013:1	Sexual offspring	F1 & M1	190	194	241	245	222	272	238	246	240	256	299	339	372	400	232	246	225	231
2013:2	Sexual offspring	F1 & M1	190	192	241	245	250	272	238	246	240	256	307	335	368	400	232	240	225	231
2013:3	Sexual offspring	F1 & M1	190	192	229	245	222	238	238	246	240	256	299	307	372	380	232	246	225	231
2015:1	Parthenogenetic offspring	F1	194	194	229	229	238	238	248	248	244	256	299	299	380	380	246	246	231	231
2015:2	Parthenogenetic offspring	F1	192	192	241	241	272	272	248	248	256	256	335	335	400	400	240	240	231	231
2015:3	Parthenogenetic offspring	F1	194	194	229	229	238	238	246	246	244	256	335	335	400	400	246	246	231	231
2015:4	Parthenogenetic offspring	F1	194	194	229	229	238	238	246	246	244	256	335	335	400	400	240	240	231	231
2016:1	Parthenogenetic offspring	F1	192	192	241	241	272	272	248	248	244	244	299	299	400	400	246	246	231	231
2016:2	Parthenogenetic offspring	F1	192	192	241	241	272	272	248	248	256	256	335	335	380	380	246	246	231	231
2016:3	Parthenogenetic offspring	F1	194	194	241	241	272	272	246	246	244	244	335	335	400	400	246	246	231	231
2016:4	Parthenogenetic offspring	F1	194	194	229	229	238	238	246	246	244	244	335	335	400	400	246	246	231	231
2016:5	Parthenogenetic offspring	F2	194	194	229	229	238	238	242	242	256	256	299	299	380	380	240	240	231	231

Genotypes are presented as base pair sizes. The mother shark *F1* is presented first, followed by the putative sire *M1* and the sexually produced adult offspring *F2*. The three deceased juvenile sharks from the final sexual breeding encounter are shown with the date 2013:1–3. The parthenogenetic offspring from *F1* are shown with the dates 2015:1–3 and 2016:1–4. The parthenogenetic offspring from *F2* is shown in row 2016:5. Ind. = individual.
