# Corrigendum: Switch from sexual to parthenogenetic reproduction in a zebra shark

**DOI:** 10.1038/srep45881

**Published:** 2017-04-07

**Authors:** Christine L. Dudgeon, Laura Coulton, Ren Bone, Jennifer R. Ovenden, Severine Thomas

Scientific Reports 7: Article number: 40537; 10.1038/srep40537 published online: 01 16 2017; updated: 04 07 2017.

This Article contains errors in Table 1. The correct Table 1 appears below as Table 1.

## Figures and Tables

**Table 1:** Genotype data at nine microsatellite loci for 15 zebra sharks *Stegostoma fasciatum* from Reef HQ Aquarium Australia. Genotypes are presented as base pair sizes.

Ind.	Description	Parent/s	SF2		SF38		SF72		Sfa221		Sfa236		Sfa248		Sfa335		Sfa387		Sfa418	
F1	Mother		192	194	229	241	238	272	246	248	244	256	299	335	380	400	240	246	231	231
M1	Father		190	190	245	245	222	250	238	242	228	240	307	339	368	372	232	232	225	225
F2 (2009)	Sexual offspring	F1 & M1	190	194	229	245	222	238	242	248	240	256	299	307	368	380	232	240	225	231
2013:1	Sexual offspring	F1 & M1	190	194	241	245	222	272	238	246	240	256	299	339	372	400	232	246	225	231
2013:2	Sexual offspring	F1 & M1	190	194	241	245	250	272	238	246	240	256	307	335	368	400	232	240	225	231
2013:3	Sexual offspring	F1 & M1	190	192	229	245	222	238	238	246	240	256	299	307	372	380	232	246	225	231
2015:1	Parthenogenetic offspring	F1	194	194	229	229	238	238	248	248	256	256	299	299	380	380	246	246	231	231
2015:2	Parthenogenetic offspring	F1	192	192	241	241	272	272	248	248	256	256	335	335	400	400	240	240	231	231
2015:3	Parthenogenetic offspring	F1	194	194	229	229	238	238	246	246	256	256	335	335	400	400	246	246	231	231
2015:4	Parthenogenetic offspring	F1	194	194	229	229	238	238	246	246	256	256	335	335	400	400	240	240	231	231
2016:1	Parthenogenetic offspring	F1	192	192	241	241	272	272	248	248	244	244	299	299	400	400	246	246	231	231
2016:2	Parthenogenetic offspring	F1	192	192	241	241	272	272	248	248	256	256	335	335	380	380	246	246	231	231
2016:3	Parthenogenetic offspring	F1	194	194	241	241	272	272	246	246	244	244	335	335	400	400	246	246	231	231
2016:4	Parthenogenetic offspring	F1	194	194	229	229	238	238	246	246	244	244	335	335	400	400	246	246	231	231
2016:5	Parthenogenetic offspring	F2	194	194	229	229	238	238	242	242	256	256	299	299	380	380	240	240	231	231

The mother shark *F1* is presented first, followed by the putative sire *M1* and the sexually produced adult offspring *F2*. The three deceased juvenile sharks from the final sexual breeding encounter are shown with the date 2013:1–3. The parthenogenetic offspring from *F1* are shown with the dates 2015:1–4 and 2016:1–4. The parthenogenetic offspring from *F2* is shown in row 2016:5. Ind. = individual.

