# Supplementary material for: Switch from sexual to parthenogenetic reproduction in a zebra shark
Source: Sci Rep. 2017 Jan 16;7:40537. doi: 10.1038/srep40537 (PMC5238396; doi:10.1038/srep40537)
Supplement: Supplementary Table [file srep40537-s1.pdf]

Switch from sexual to parthenogenetic reproduction in a zebra shark

Christine L Dudgeon[1 *]

Laura Coulton[2]

Ren Bone[2]

Jennifer R Ovenden[1]

Severine Thomas[2,3]

[1]The University of Queensland, Molecular Fisheries Laboratory, School of Biomedical Sciences, St. Lucia Queensland, 4072, Australia.

[2]Reef HQ Aquarium, Townsville, Australia.

[3]College of Marine and Environmental Sciences, James Cook University, Townsville, 4811, Queensland, Australia

[*]Corresponding Author

Email: c.dudgeon@uq.edu.au

Supplementary Table. The mother shark F1 is presented first, followed by the putative sire M1 and the sexually produced adult offspring F2. The three deceased juvenile sharks from the final sexual breeding encounter are shown with the date 2013:1 – 3. The parthenogenetic offspring from F1 are shown with the dates 2015:1-3 and 2016:1-4. The parthenogenetic offspring from F2 is shown in row 2016:5. Ind. = individual.

**Ind.**	**Description**	**Parent/s**	SF41		SF138		Sfa205		Sfa382		Sfa454	
F1	Mother		208	222	176	178	360	372	174	182	189	201
M1	Father		208	220	176	180	356	360	182	186	199	201
F2 (2009)	Sexual offspring	F1 & M1	220	222	176	180	356	372	174	182	189	199
2013:1	Sexual offspring	F1 & M1	208	208	178	180	356	372	182	186	189	201
2013:2	Sexual offspring	F1 & M1	208	222	176	180	356	372	182	186	201	'201
2013:3	Sexual offspring	F1 & M1	208	220	176	180	356	372	182	182	189	201
2015:1	Parthenogenetic offspring	F1	208	208	178	178	372	372	174	174	201	201
2015:2	Parthenogenetic offspring	F1	208	208	178	178	372	372	182	182	201	201
2015:3	Parthenogenetic offspring	F1	208	208	176	176	372	372	174	174	189	189
2015:4	Parthenogenetic offspring	F1	208	208	178	178	372	372	182	182	189	189
2016:1	Parthenogenetic offspring	F1	222	222	178	178	360	360	174	174	189	189
2016:2	Parthenogenetic offspring	F1	222	222	178	178	372	372	182	182	201	201
2016:3	Parthenogenetic offspring	F1	208	208	178	178	360	360	182	182	189	189
2016:4	Parthenogenetic offspring	F1	222	222	178	178	360	360	182	182	201	201
2016:5	Parthenogenetic offspring	F2	222	222	176	176	372	372	182	182	189	189
